# Improving robustness of automatic cardiac function quantification from cine magnetic resonance imaging using synthetic image data

**DOI:** 10.1038/s41598-022-06315-3

**Published:** 2022-02-14

**Authors:** Bogdan A. Gheorghiță, Lucian M. Itu, Puneet Sharma, Constantin Suciu, Jens Wetzl, Christian Geppert, Mohamed Ali Asik Ali, Aaron M. Lee, Stefan K. Piechnik, Stefan Neubauer, Steffen E. Petersen, Jeanette Schulz-Menger, Teodora Chițiboi

**Affiliations:** 1 Advanta, Siemens SRL, Brașov, Romania; 2 Systems Engineering, Transilvania University of Brașov, Brașov, Romania; 3 Digital Technology and Innovation, Siemens Healthineers, Princeton, NJ USA; 4 Magnetic Resonance, Siemens Healthineers, Erlangen, Germany; 5 Digital Technology and Innovation, Siemens Healthineers, Bangalore, India; 6 Division of Cardiovascular Medicine, Radcliffe Department of Medicine, University of Oxford, Oxford, UK; 7 William Harvey Research Institute, NIHR Biomedical Research Centre at Barts, Queen Mary University of London, London, UK; 8 Barts Heart Centre, St Bartholomew’s Hospital, Barts Health NHS Trust, West Smithfield, London, UK; 9 Health Data Research UK, London, UK; 10 The Alan Turing Institute, London, UK; 11 Charité-Universitätsmedizin Berlin, Experimental and Clinical Research Center, Working Group On CMR and HELIOS Klinikum Berlin Buch, Cardiology Berlin, DZHK partnersite Berlin, Berlin, Germany

**Keywords:** Computer science, Cardiology

## Abstract

Although having been the subject of intense research over the years, cardiac function quantification from MRI is still not a fully automatic process in the clinical practice. This is partly due to the shortage of training data covering all relevant cardiovascular disease phenotypes. We propose to synthetically generate short axis CINE MRI using a generative adversarial model to expand the available data sets that consist of predominantly healthy subjects to include more cases with reduced ejection fraction. We introduce a deep learning convolutional neural network (CNN) to predict the end-diastolic volume, end-systolic volume, and implicitly the ejection fraction from cardiac MRI without explicit segmentation. The left ventricle volume predictions were compared to the ground truth values, showing superior accuracy compared to state-of-the-art segmentation methods. We show that using synthetic data generated for pre-training a CNN significantly improves the prediction compared to only using the limited amount of available data, when the training set is imbalanced.

## Introduction

Cardiovascular disease is the leading cause of death globally, according to the World Health Organization. Cardiovascular magnetic resonance imaging (MRI) is considered the gold standard for evaluating heart function. Estimating the ventricular end-systolic (ESV) and end-diastolic (EDV) volumes, stroke volume (SV) and ejection fraction (EF) from cardiac MRI is a prerequisite for assessing cardiovascular diseases, and typically requires careful and precise contouring of the ventricles.

Deep learning (DL) is predicted to bring substantial change to how cardiovascular MRI is acquired and analyzed^1^. The gradual adoption of DL to solve medical image analysis tasks has spawned hundreds of articles addressing the automatic segmentation of cardiac chambers from MRI^2^, including several segmentation challenges organized by societies such as MICCAI^3^ and Kaggle^4^. For example, Bai et al.^5^ proposed a deep learning segmentation approach using a fully convolutional network (FCN). Liao et al.^6^ also proposed a deep learning segmentation approach using a modified FCN called Hypercolumns Fully Convolutional Neural Network (HFCN), where features from different levels are concatenated along channel axis. DL algorithms are increasing their performance thanks to the larger annotated datasets available, such as the UK Biobank^7^, but data with ground-truth segmentations is typically not sufficiently representative of cardiovascular disease phenotypes, scanners, sequences, and protocols, which limits generalizability. Moreover, experts do not always agree on the precise contour location, as captured by the reduced inter-observer reproducibility of manual contours^8^, and corrections are still routinely required^3^.

Data augmentation is routinely used in training DL models for medical imaging to increase and diversify the training data set but is often limited to affine transformations and noise addition, which cannot generate cases with diverse clinical and scan parameters. In recent years, there has been a growing interest in DL for synthetic data generation, notably starting with Generative Adversarial Networks (GAN)^9^ which can map a random noise vector to a synthetically generated image. A major disadvantage of GAN is the lack of control over the generated images, which was mitigated with the introduction of conditional GANs^10^. Style transfer DL architectures (CycleGAN^11^, Pix2Pix^12^) convert an input image from one domain to another, by modifying the style, while preserving the content. Unsupervised style transfer has been applied from standard CINE MRI to LGE^13^ and CT^14^, but with limited application to cardiovascular pathologies. The main drawback of style transfer is the need for a large set of annotated images from at least one domain, that is representative of all cardiac anatomy phenotypes. Semantic image synthesis approaches (mask-to-image translation) map one or more segmentation masks to a corresponding image, i.e. the opposite of segmentation networks. GauGAN^15^ is a novel approach using a Spatially Adaptive Normalization (SPADE)^16^ technique which is a combination between batch normalization and instance normalization, implemented as a two-layer CNN. The network produces a realistic, completely new images, thus introducing more shape, texture, and background variations than conventional computer vision-based augmentation techniques. In one cardiac MRI application, Abbasi-Sureshjani et al.^17^ have used a GauGAN network to synthesize labeled 3D + t CINE images. The usage of synthetic data has been previously shown to improve deep-learning based segmentation models, when little training data is available^18^.

Other AI approaches focus on direct cardiac function quantification though regression, without producing an aggregated segmentation of the structure of interest. Luo et al.^19^ proposed a DL regression approach based on a multi-scale atlas for the left ventricle (LV) location and a deep Convolutional Neural Network (CNN). One benefit of regression methods is that they can incorporate training data where only the EDV and ESV values are available, e.g., from a radiology report, without requiring ground-truth segmentation masks, which are challenging and costly to obtain.

In this work, we investigate the automatic cardiac function quantification as a regression task. Our first contribution is a Residual Spatial Feature Encoding Recurrent network for Abstracting high-level patient features (SFERA) to predict left ventricle volumes (and implicitly the EF) without explicit segmentation. The network combines a fully convolutional feature encoder that learns the cardiac geometry with a recurrent network based on a bidirectional LSTM^20^ that incorporates the volumetric information over a stack of variable number of short-axis slices. To train our proposed regression network, a large dataset with a wide and dense distribution of ground truth EDV and ESV values would be required to ensure an accurate and robust performance across the entire continuum of values. We hypothesize that synthetically generated cardiac MRI can substantially improve the performance of our regression model. To show this, our second contribution is a DL approach based on the GauGAN^15^ architecture, to synthetically generate short axis (SAX) cardiac MRI stacks with a wide range of EF values, to be more representative of real-world clinical cases. The SFERA network was pre-trained on the large synthetically generated dataset, and then finetuned on real cases. Our final EF prediction error is comparable or slightly smaller than other state-of-the-art methods.

## Results

### Synthetic image generation

**Figure 1:**
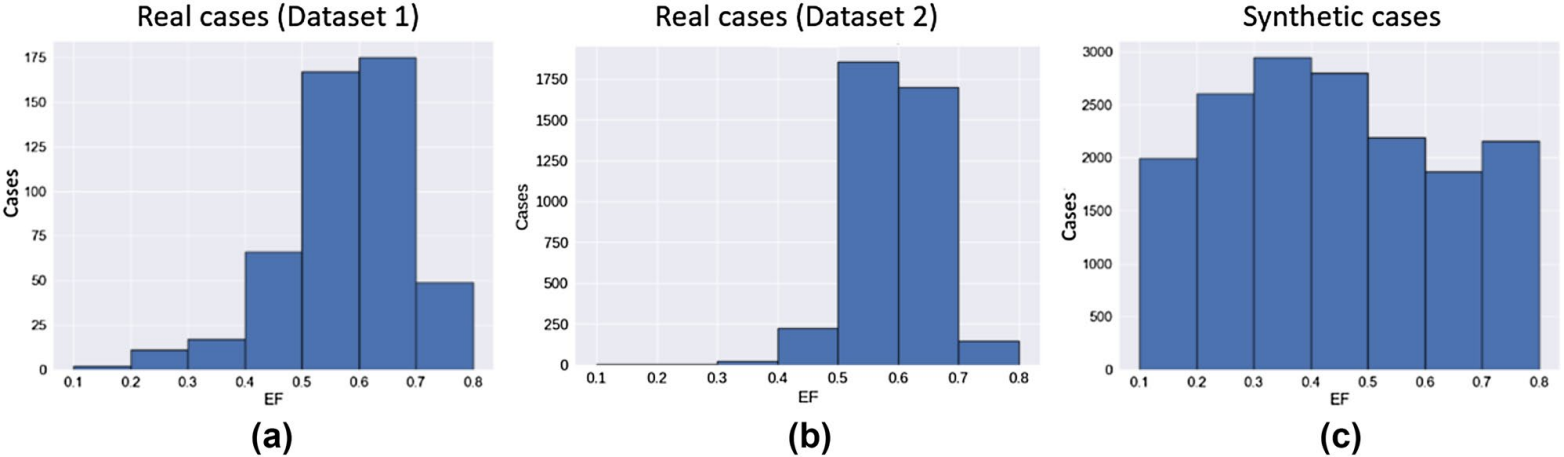
Normal EF distributions of the 491 real cases from the Dataset 1 (**a**) and 3975 cases from Dataset 2 (**b**), and our 22,861 synthetically generated cases with a uniform EF distribution (**c**).

**Figure 2:**
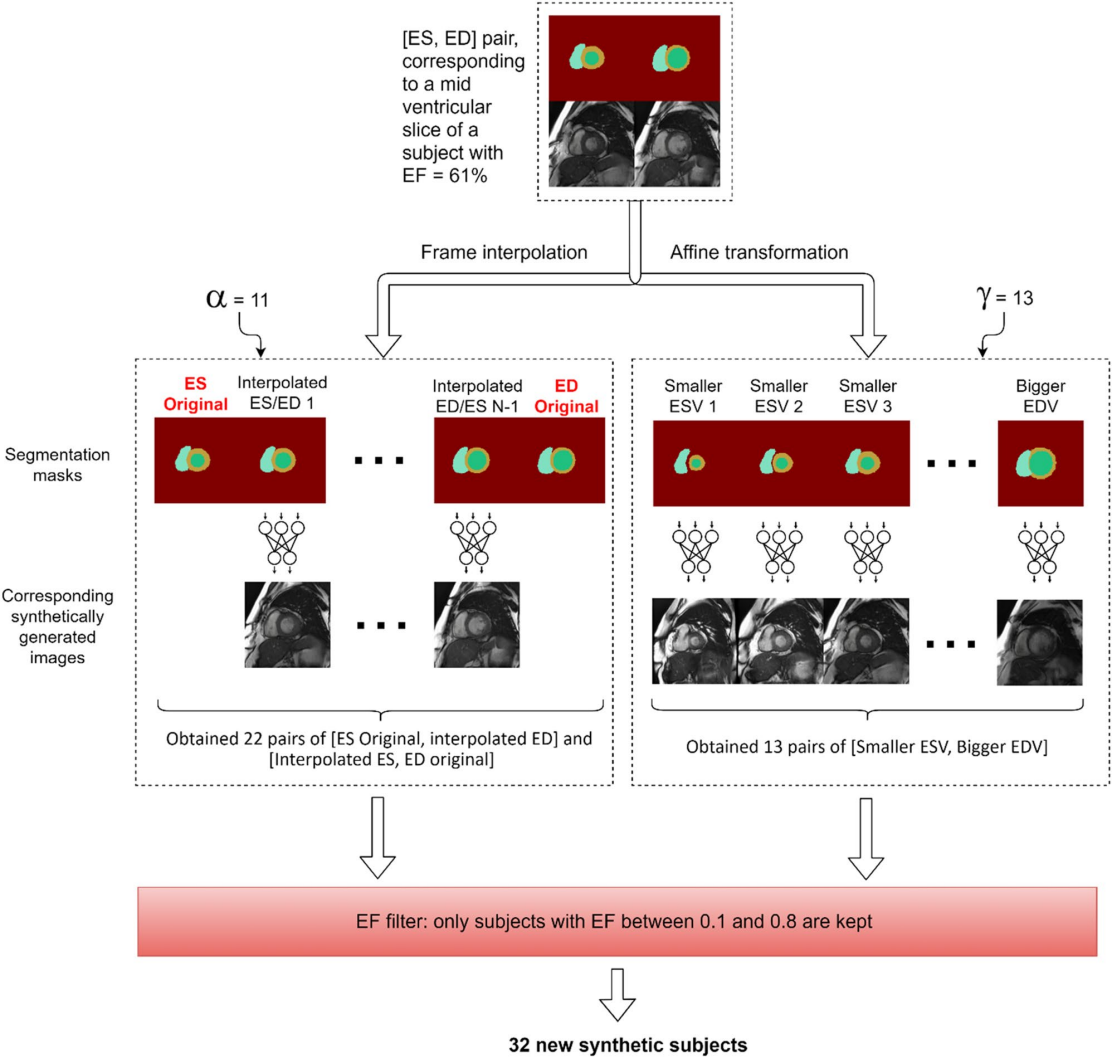
Workflow for the synthetic image generation step. The end-systolic and end-diastolic frames from every slice of the training data goes through this process to generate an extended set of masks with different EF values. Parameters α and $\gamma$ are used to control the number of interpolated frames between [ES, ED], and the number of rescaled [ES, ED] pairs. In order to generate a new synthetic slice (ed, es pair) with smaller EF, new pairs of [ES original, Interpolated ED] and [Interpolated ES, ED original] are chosen. To generate a new synthetic slice with higher EF, a pair of [Smaller ESV, Bigger EDV] is chosen. An additional step is employed to filter only resulting subjects with EF between 10 and 80%.

**Figure 3:**
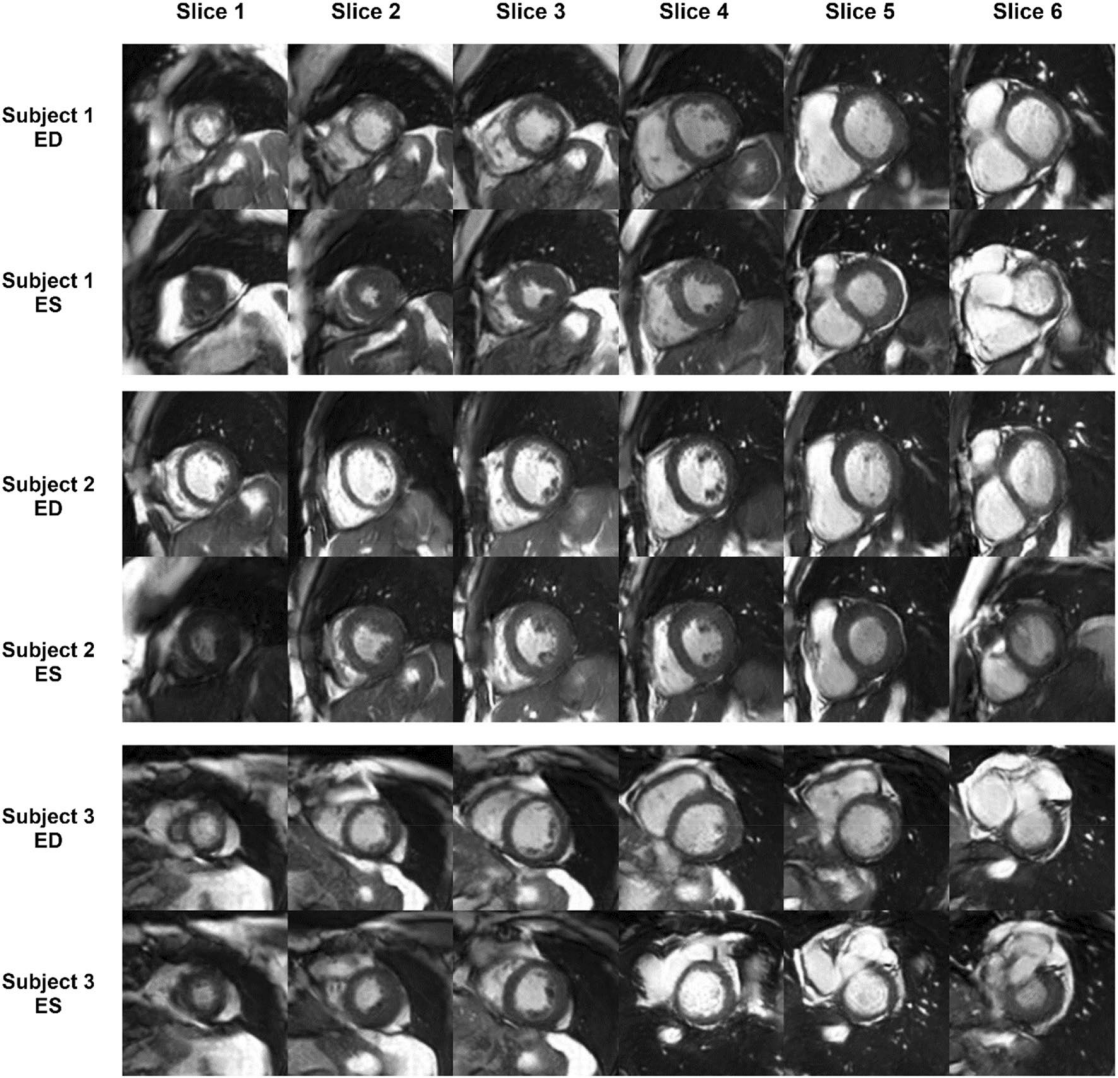
Examples of three synthetically generated SAX stacks at ED and ES.

Figure 1a,b shows the normal distribution of the EF parameter in the two large datasets. In the original datasets, the reported EF was reduced (< 40%) in only 6.3% of the cases and high (> 70%) in only 10.5% of the cases. For a small to moderate training data size, this data imbalance can lead to suboptimal results for the pathological cases, i.e. an AI algorithm trained on such data distributions may perform poorly on the less represented low or high EF cases. Hence, by automatically processing the segmentation masks of our real training subjects, we synthetically generated 22,653 new SAX stacks consisting of ED and ES masks for the left and right ventricles with a uniform distribution along the LV EF spectrum as shown in Fig. 1c. Using a deep-learning network adversarial-trained for real patient data for mask-to-image generation, the synthetic masks were used to generate the same number of synthetic cardiac MR subject datasets. Figure 2 shows the entire workflow for generating new synthetic slices with a wide range of EF values, starting from a mid-ventricular slice of a real subject, as an example. For more details see the “Methods” section. The resulting synthetic cohort was approximately 32 × larger than the real subject cohort. Figure 3 shows three example synthetic subjects generated using the proposed approach.

### Cardiac function prediction

The baseline results, obtained by training our proposed SFERA network for cardiac function prediction solely on real case data with a normal EF distribution are referred to as *Real Subjects Only* (RSO). The same network architecture trained entirely on synthetic data with a uniform EF distribution is referred to as *Synthetic Subjects Only* (SSO). The SSO model finetuned on real cases is referred to as *Real Subjects with Pretraining* (RSP).

The *Real Subjects All* (RSA) experiment represents the same network architecture, but trained only on real data from both datasets (without finetuning).

**Figure 4:**
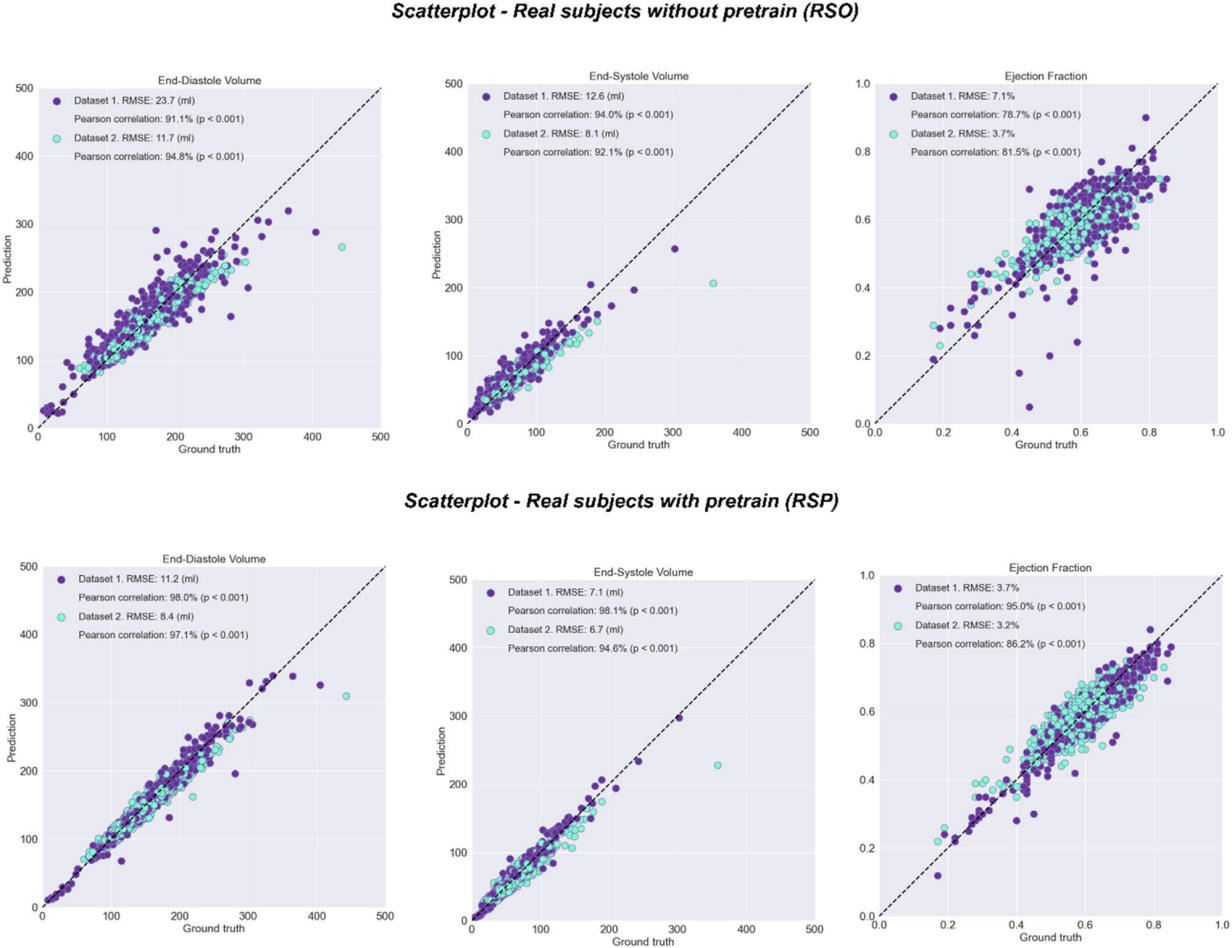
Scatter plots of predicted and ground truth volumes and EF on the Dataset 1 test dataset (purple) and Dataset 2 test dataset (light blue), for the model trained on real cases only, without pretraining (RSO) and the model finetuned on real cases after pretraining on synthetic data (RSP). RMS Error is computed for EDV, ESV and ejection fraction.

Figure 4 shows the correlation between the manually annotated and the automatically predicted LV volumes and EF for the models with and without pretraining. The Pearson correlation values corresponding to RSO experiment (without pretraining) for EF, EDV and ESV are 78.7%, 91.1% and 94.0% ($p$ < 0.001) for Dataset 1 and 81.5%, 94.8%, 92.1% ($p$ < 0.001) for Dataset 2, as shown in Fig. 4 top. In the RSP experiment (with pretraining), the Pearson correlation values for EF, EDV and ESV increased to 95.0%, 98.0% and 98.1% ($p$ < 0.001) for Dataset 1, and 86.2%, 97.1%, 94.6% ($p$ < 0.001) for Dataset 2, as shown in Fig. 4 bottom.

**Figure 5:**
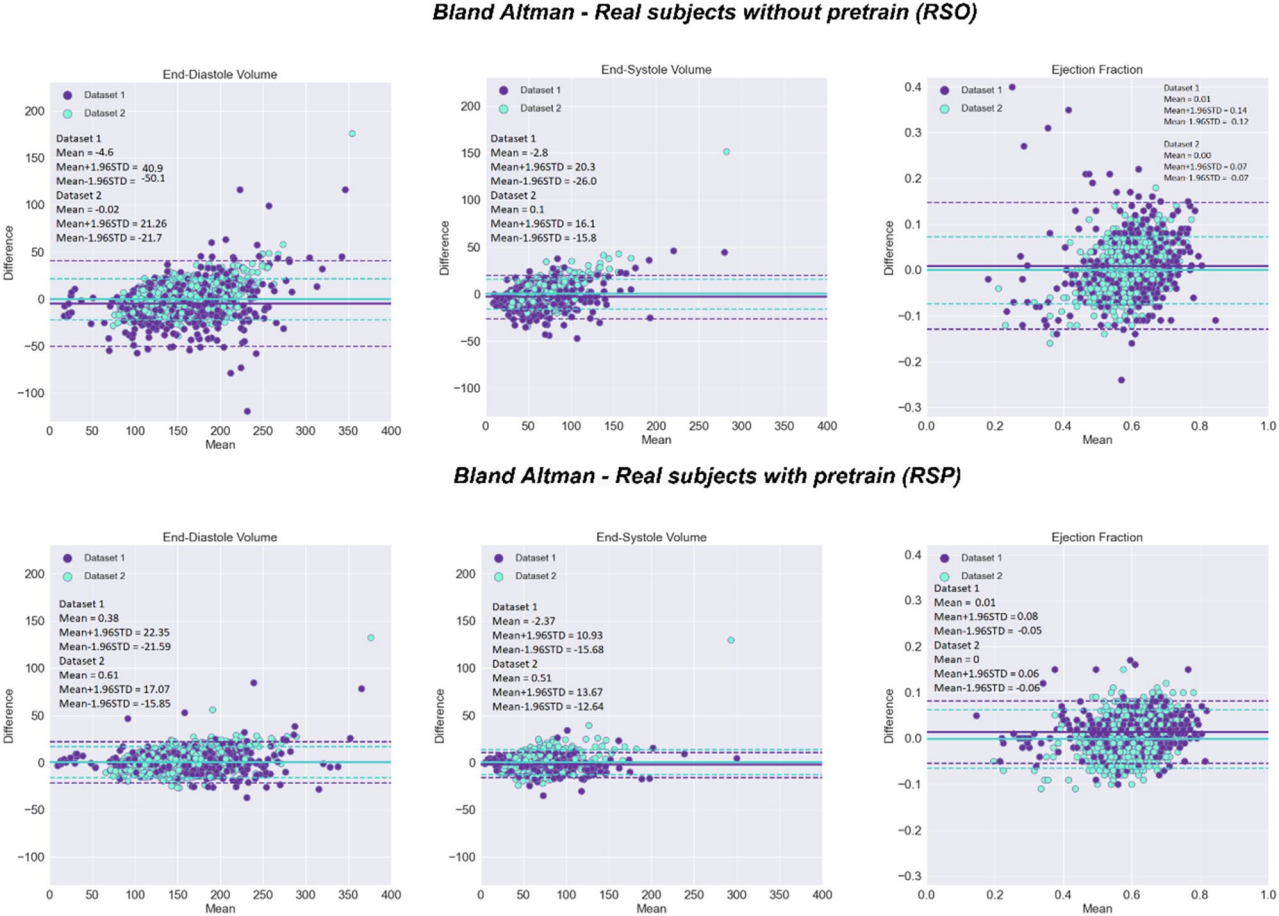
Bland–Altman (BA) plots of predicted and ground truth volumes and EF on the Dataset 1 test dataset (purple) and Dataset 2 test dataset (light blue), for the model trained on real cases only, without pretraining (RSO) and the model finetuned on real cases after pretraining on synthetic data (RSP). Bland–Altman (BA) analysis of the results, comparing the models trained on real cases without pretraining (top) and real cases with finetuning from the synthetic model (bottom).

The Fig. 5 shows the Bland–Altman analysis for the volumes and the EF predictions on our two test sets, for the experiments trained on real cases without and with pretraining. In both cases no bias was observed. The mean RMS error in the RSO experiment for the EF was 7.1% for Dataset 1 and 3.7% for Dataset 2. In the RSP experiment, the root mean squared error (RMSE) was significantly reduced to 3.7% for Dataset 1 and 3.2% for Dataset 2 ($p$ < 0.005). Similarly, the RMSE was significantly reduced from 23.7 to 11.2 ml ($p$ < 0.005) for Dataset 1 and from 11.0 to 8.4 ml for Dataset 2 for EDV. For ESV, the RMSE was reduced from 12.6 to 7.9 ml ($p$ < 0.005) for Dataset 1 and from 8.1 to 6.7 ml for Dataset 2.

The mean absolute error (MAE) in the RSO experiment for the EF was 4.9% for Dataset 1 and 2.8% for Dataset 2. In the RSP experiment, the mean absolute error was significantly reduced to 2.7% for Dataset 1 and 2.5% for Dataset 2 ($p$ < 0.005). Similarly, the mean absolute error was significantly reduced from 16.8 to 7.3 ml ($p$ < 0.005) for Dataset 1 and from 8.0 to 6.2 ml for Dataset 2 for EDV. For ESV, the mean absolute error was reduced from 9.0 to 5.2 ml ($p$ < 0.005) for Dataset 1 and from 5.6 to 4.7 ml for Dataset 2.The 95% confidence intervals of the MAE for EF, computed using bootstrapping, are [2.0, 2.1] in SSO experiment, [4.4, 5.3] and [2.7, 2.9] for RSO experiment Dataset 1 and Dataset 2, [2.4, 2.9] and [2.4, 2.6], for RSP experiment, Dataset 1 and Dataset 2.

Table 1 compares the RMSE of EDV, ESV, and EF prediction, for the RSO, RSA, and RSP experiments with our proposed approach, and the results of the winning team^21^ of the Kaggle challenge (based on the mean Continuous Ranked Probability Score (CRPS)^22^ metric) and the results of the top 4^6^ team (which had the lowest RMSE for EF in the competition).Namely, the winning team Luo et al.^21^ obtained a 0.00948 CRPS^22^ score, which is the equivalent of 12.0 ml RMS error for EDV, 10.2 ml for ESV and 4.9% ejection fraction. The smallest ejection fraction error, 4.7 was obtained by the top 4 team Liao et al.^6^, even though the RMSE for volumes is a slightly bigger. We also compared our results with a previously published state-of-the-art approach on the Dataset 2^5^.

**Table 1:** RMSE ± SD for the EDV, ESV and EF prediction from top to bottom for our models trained on synthetic subjects only (SSO), real subjects only (RSO), real subjects all (RSA) experiment which means trained on all real subjects from combined datasets and SSO model finetuned on real cases is referred to as real subjects with pretraining (RSP). RSP Below are the results of the winner of the Kaggle challenge^4^ (based on the mean CRPS^22^ metric), the results of the top 4 team^6^ (which had the lowest RMSE for EF in the competition), and previously reported results on the Dataset 2^5^ for comparison.

Experiment	RMS error					
	EDV (ml) Dataset1	ESV (ml) Dataset1	EF (%) Dataset1	EDV (ml) Dataset2	ESV (ml) Dataset2	EF (%) Dataset2
SSO (ours)	56.6	36.1	8.0	–	–	–
RSO (ours)	23.7	12.6	7.1	11.7	8.1	3.7
RSA (ours)	13.3	9.6	6.6	9.2	7.3	3.5
RSP (ours)	**11.2**	**7.1**	**3.7**	**8.4**	**6.7**	**3.2**
Top1 Kaggle^4^	12.2	10.1	4.8	–	–	–
Top4 Kaggle^6^	13.2	9.3	4.6	–	–	–
Bai et al.^5^	–	–	**–**	6.1 ± 5.3	5.3 ± 4.9	3.2 ± 2.9

Significant values are in bold.

We additionally show that while a large pretraining dataset improves the prediction, the potential for improvement is bounded. We could reach a similar accuracy using only a random 50% of the available synthetic data (RMSE 3.8) compared to using the full dataset (RMSE 3.7). Selecting 50% of our synthetic data such that it has the same distribution as the original Dataset 1 lead to a similar result (RMSE 3.9). However, when considering only the test subjects with a reduced EF < 40%, the model pretrained on synthetic data with a normal distribution of the EF parameter had a lower error compared to the model pretrained on data with the same EF distribution as the original Dataset 1 (RMSE 3.0 vs. 4.2).

The inference time on a desktop computer with the following hardware configuration: Intel^®^ Core™ i7-7700 K CPU @ 4.20 GHz, NVIDIA GeForce GTX 1080 Ti graphics card, 64 GB RAM was around 5.5 ± 4.3 ms.

## Discussion

Our initial RSO model trained only on real data is not able to reach the same performance of state-of-the-art DL segmentation approaches on the same dataset. By addressing the automatic cardiac volume computation as a regression task, we are introducing more sensitivity to the distribution of the cardiac volumes over the training data, than in a classic image segmentation based setting. We observed that having a wide and dense distribution of values in the training set is crucial for achieving good accuracy across the entire range of values.

Our RSP model, first pretrained on synthetic data, by far outperforms the baseline RSO model trained only on real data. The EF prediction error decreases significantly when synthetic data is used for pretraining. Similarly, the Pearson correlation for the EDV, ESV, and EF is significantly higher for RSP compared to RSO. Pre-training has a high impact especially for cases with low or very high EF values, which had a low density in the initial distribution.

The RSA model, which was jointly trained on Dataset 1 and Dataset 2 and evaluated on the two test sets, has an improved performance compared to the RSO model, indicating that having more data overall improves the results. However, since combining the datasets does not lead to a wide and dense distribution of the ejection fraction values, the performance is inferior when compared to the RSP scenario where synthetic data with a quasi-uniform ground truth value distribution is employed for pre-training. Hence, performing pretraining on a large dataset where the EF is uniformly distributed is preferred to using a large dataset that preserves the EF imbalance of the original data.

Our final prediction model after pretraining on synthetic data (RSP) performs well compared to other state-of-the-art approaches. Since the original ground-truth of the Kaggle challenge test set is not publicly available, our results on Dataset 1 were based on our own manual segmentation of the CINE MRI data, so they are not directly comparable to the Kaggle challenge results. Nevertheless, our model shows very promising performance emphasized by a tight confidence interval.

A main benefit of our first contribution, the SFERA network for determining the EDV and ESV through regression, is that we can use training data where only the cardiac volumes and ejection fraction are provided as ground truth, without the need for a segmentation mask. Finetuning the network on a new dataset acquired with a different scanner, imaging protocol, or including new pathologies is often necessary when adapting a DL model to routine clinical data. In this setting, the EDV and ESV values could be more easily obtained in practice, for example from a radiology report, compared to full segmentation contours. More specifically, when finetuning on Dataset 2, our network only uses the EDV and ESV values. Nevertheless, our performance is close to a state-of-the-art segmentation approach trained on the segmentation masks. The main reason why the performance of the SFERA model does not improve more after pretraining on synthetic data is that Dataset 2 contains mostly healthy subjects, with an ejection fraction in the range 50–60%. Thus, adding synthetic data from a wider range of ejection fractions in this case does not have such a large positive impact overall.

**Figure 6:**
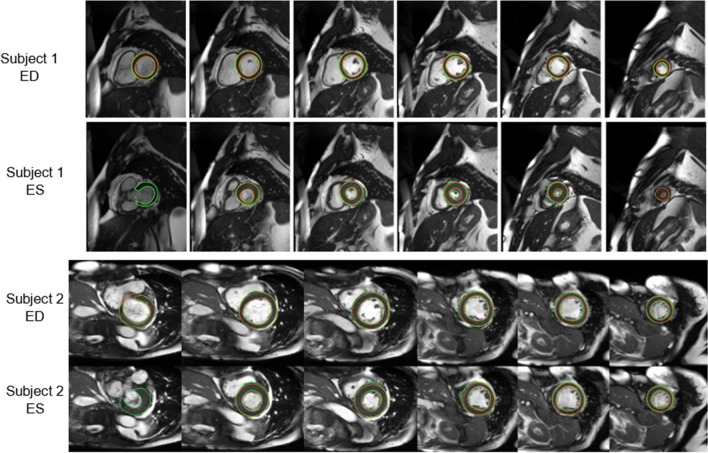
Examples EF prediction using the proposed methods compared to manual segmentation (green) and automatic segmentation (orange). Subject 1—top: Annotated EF = 66%; AutoSegmented EF = 76%; Predicted EF (proposed method) = 70%. Subject 2—bottom: Annotated EF = 32%; AutoSegmented EF = 42%; Predicted EF = 31%. Ground truth contours in green, Segmented contours in orange. Some slices are omitted (with similar contour quality).

The main disadvantage of our first contribution is that the result of the SFERA network is more difficult to confirm without the contours present, compared to a segmentation network. However, regression approaches could potentially serve as a verification step for a segmentation network, to help increase confidence in the final measurement when dealing with uncertainty. Another potential application is to filter out normal cases that do not require further precise quantification, which could save reading time. Hybrid approaches may employ an ensemble that combine different segmentation and regression solutions to improve the accuracy of the combined result^23^. For example, depending on how the basal slices are subjectively handled in manual vs. automatic contouring, segmentation-based approaches may introduce notable differenced in the EF in some cases. Figure 6 shows two sample subjects from Dataset 1 with overlaid manually annotated and automatic contours obtained using a state-of-the-art cardiac segmentation prototype^24^. For both subjects, predicted EF values using the proposed method (70% and 31% respectively) are similar to the EF values computed based on the manually segmented contours (66% and 32% respectively). The automatic segmentation algorithm inaccurately segments the base and apex at ES, and therefore the EF predictions obtained with the proposed approach is closer to the ground truth compared to the EF obtained by automatic segmentation (76% and 42% respectively).

An advantage of our second contribution, namely the image synthesis approach, is that we are able to generate realistic-looking cardiac anatomy including papillary muscles and trabeculations inside the blood pool, which could then be used for pre-training. The synthetic data may also include small image artefacts, different image sharpness and varying contrast, similar to the original dataset used for training, which contribute to the realistic aspect. These synthetic cases thus reliably serve in the pre-training step for the ventricle volume and EF prediction task.

One limitation of our image synthesis approach is that the network was trained on individual 2D frames. This causes the image background to be somewhat inconsistent between ED and ES and for consecutive slices of the same case. As shown in Fig. 3, the background may not always be anatomically accurate because no segmentation of the background structures was included when training the GauGan network. Nevertheless, the background generally captures the diaphragm, abdominal structures, lungs and chest wall, as well as the familiar texture expected from MRI, making it suitable for pretraining. In future work, we plan to extend the approach to generate consistent 3D volumes.

Our proposed image synthesis DL network also requires an initial segmentation of the training data, to generate new synthetic patients. In a novel approach, the need for manual segmentation could be circumvented by using an autoencoder^25^, one direction which we will further investigate. Another limitation is that the ED and ES frames are needed to be preselected as input to the volume prediction network. However, this task could also be performed by an independent neural network trained to automatically identify ED and ES timepoints from a CINE series such as^26^.

In general, while Dataset 2 contains mostly healthy subjects, the Dataset 1 data does contain some examples of unspecified cardiovascular pathologies but the precise disease labeling has not been made publicly available. However, this data is still not sufficiently representative of commonly imaged cardiovascular diseases such as: cardiomyopathies, dyssynchrony, akinetic or dyskinetic wall segments, or apical aneurysms. Our proposed image synthesis network could, in principle, be trained on data where such pathologies are well represented to produce more diverse synthetic cases.

In conclusion, we showed that generating synthetic training data with machine learning can be a powerful tool for improving results of deep learning pipelines, especially when only unbalanced, scarce data is available. In this work, we considered the task of automatically predicting the ventricle volumes from Cardiac MRI as a regression problem and we proposed a custom regression network (SFERA) to tackle this challenge. We have demonstrated that pretraining on a large synthetic dataset with a uniform distribution of the ejection fraction greatly improves the prediction compared to only using the limited amount of available data. To show this, we devised a two-step methodology: first, we generate synthetic data with a uniform distribution of EF values, by using a computer vision-based algorithm for generating binary masks and adopting a mask-to-image network. In the second phase, we pre-trained a neural network only on synthetic data, then finetuned it on the real cases. This methodology was demonstrated using two different datasets, with accurate results compared to the state-of-the-art. The same image synthesis approach is generalizable to other medical image analysis tasks where the distribution of the available training data is insufficiently representative, or the amount of data is scarce.

## Methods

### Data

The Kaggle Data Science Bowl Cardiac Challenge Data^4^ [Dataset 1] consists of CINE bSSFP cardiac MRI including a short-axis (SAX) stack which was used for ventricular volume quantification. This dataset is publicly available^4^. The data was acquired with 8–10 mm slice thickness, spatial resolution between 0.61–1.95 mm × 0.61–1.95 mm, and approximately 30 cardiac frames per slice, at 1.5 and 3 T (MAGNETOM Aera and Skyra, Siemens Healthcare, Erlangen, Germany). The average distance between consecutive SAX slices was 9.8 ± 0.6. Since the segmentation masks used to generate the EDV and ESV values used as ground truth in the competition were not made publicly available, the entire dataset was re-annotated by an expert observer. All individual ED and ES frames were manually identified, and the LV and right ventricle (RV) were manually contoured. The annotations were used to compute ground truth values for the ED and ES LV volumes. The subjects with less than 5 consecutive SAX slices or with the presence of significant motion artefacts were excluded from the training and validation subsets. 491 subject datasets were used for training, 187 for validation and the remaining 440 (same test set as in the original challenge) were reserved for testing.

A second independent dataset was publicly available from the UK Biobank Resource^7^ [Dataset 2]. CINE bSSFP cardiac MR data was acquired using a standard protocol^27^. The SAX stack spanning from the apex to the base of the left ventricle was acquired with 8 mm slice thickness, a spatial resolution ranging between 1.8–2.1 mm × 1.8–2.1 mm, and a 31 ms temporal resolution at 1.5 T (MAGNETOM Aera, Siemens Healthcare, Erlangen, Germany). The average slice distance was 8.89 ± 0.88 mm. A ground truth annotation of the LV and RV was obtained through manual segmentation of the end-systolic (ES) and end-diastolic (ED) phases by an expert observer. 3975 subjects were used for training, 300 for validation and the rest of 412 were reserved for testing.

The data was resampled to 1 × 1 mm spatial resolution, cropped to 150 × 150 pixels around the image center and the image intensity values were normalized to the [3%, 97%] quantiles.

### Synthetic image generation

The right approach for synthetic data generation depends on several factors: availability of annotated data, desired quality of the synthetic data, reproducibility, and the amount of control over the characteristics of the generated data (e.g. class label, the size and deformation of the structures). Herein, we describe a semantic image synthesis algorithm, capable of fully controlling the size and location of the resulting anatomical structures to obtain synthetic subjects with different EF values.

We adapted a state-of-the-art DL network architecture for mask-to-image translation GauGAN^15^ to the task of generating synthetic ED and ES image frames of a cardiac SAX image stack, while fully controlling the volume an ejection fraction of the LV. The generator consists of multiple SPADE blocks and the discriminator is a simple convolutional neural network. The loss function is computed from three weighted terms: Multiscale Adversarial Loss and two feature matching losses (one using the discriminator and the other one using a pretrained network).

We first trained the synthetic image generation network using the training subset of Dataset 1 consisting of CINE MR images and manually annotated segmentation masks with three labels for the LV, RV, and myocardium. The network was trained using the deterministic approach introduced in Ref.^15^ where we only use the segmentation mask as input. Taesung et al. also suggest a latent space vector to adjust the appearance of the produced synthetic images. However, in our experiments, using a latent space resulted in less realistic images, so we decided to use the strictly deterministic approach. The number of epochs used to train the image synthesis model was chosen empirically based on the subjective visual assessment of the generated images.

Next, we generated an extended dataset of synthetic masks to be used as input for the GauGAN model. For this, we used as starting point the segmentation masks in the Dataset 1 training subset. First, we perform for all slices an interpolation on (ED, ES) mask pairs, and return a number of $\mathrm{F}=11$ intermediate interpolated masks computed as follows:

1 $$IM=\left(\frac\alpha F\times{SDT}_1\right)+\left(\left(1-\frac{F-\alpha}F\right)\times{SDT}_2\right)$$

where IM represents the interpolated mask, ${SDT}_{1} and {SDT}_{2}$ represent signed distance transform masks of ED and ES and $\alpha \in (0, F)$. Pairs of (ED, interpolated ED) and (interpolated ED, ES) masks are used to create synthetic cases with reduced EF. In the second step, we use an affine transformation $\gamma$ to rescale the ED and ES masks, such that anatomical structures become smaller at ES, and larger at ED. Thirteen uniformly distributed sample values of $\gamma$ over the interval [0.7, 1) are used for rescaling the ES mask, leading to a smaller LV and implicitly a smaller volume. The same number of samples are used for the ED mask, but covering the interval [1, 1.2), resulting in a larger LV for ED mask and an increased EF for the case. The values of $\mathrm{\alpha }$ and $\gamma$ have been chosen empirically.

The synthetically generated masks contained the same number of slices as the real cases used as starting point. The EDV and ESV for the synthetic subjects were computed using Simpson’s rule, assuming a constant slice thickness of 8 mm, and no gaps between slices. The EF is computed from the resulting volumes as:

2 $$EF = \frac{\left(EDV - ESV\right)}{EDV}.$$

Finally, we applied the trained image synthesis model described above to the previously generated extended set of synthetic masks with a uniform EF distribution to generate the synthetic CINE MR images. Three synthetically generated SAX stacks can be seen in Fig. 3.

The resulting 22,653 synthetic cases were split into 16,491 synthetic cases for training the SFERA network for cardiac function prediction, and 6162 for validation for the pretraining step in the RSP experiment. To assess the importance of a uniform EF distribution in the pretraining dataset, we selected a subset of 8245 synthetic cases (50% of the available pretraining data) such that the EF distribution was similar to the original Dataset 1 shown in Fig. 1. We also randomly selected another subset of 8245 cases with a uniform EF distribution. We then compared the performance of the models pretrained on these two subsets with the model pretrained on all available synthetic data.

### Cardiac function prediction

**Figure 7:**
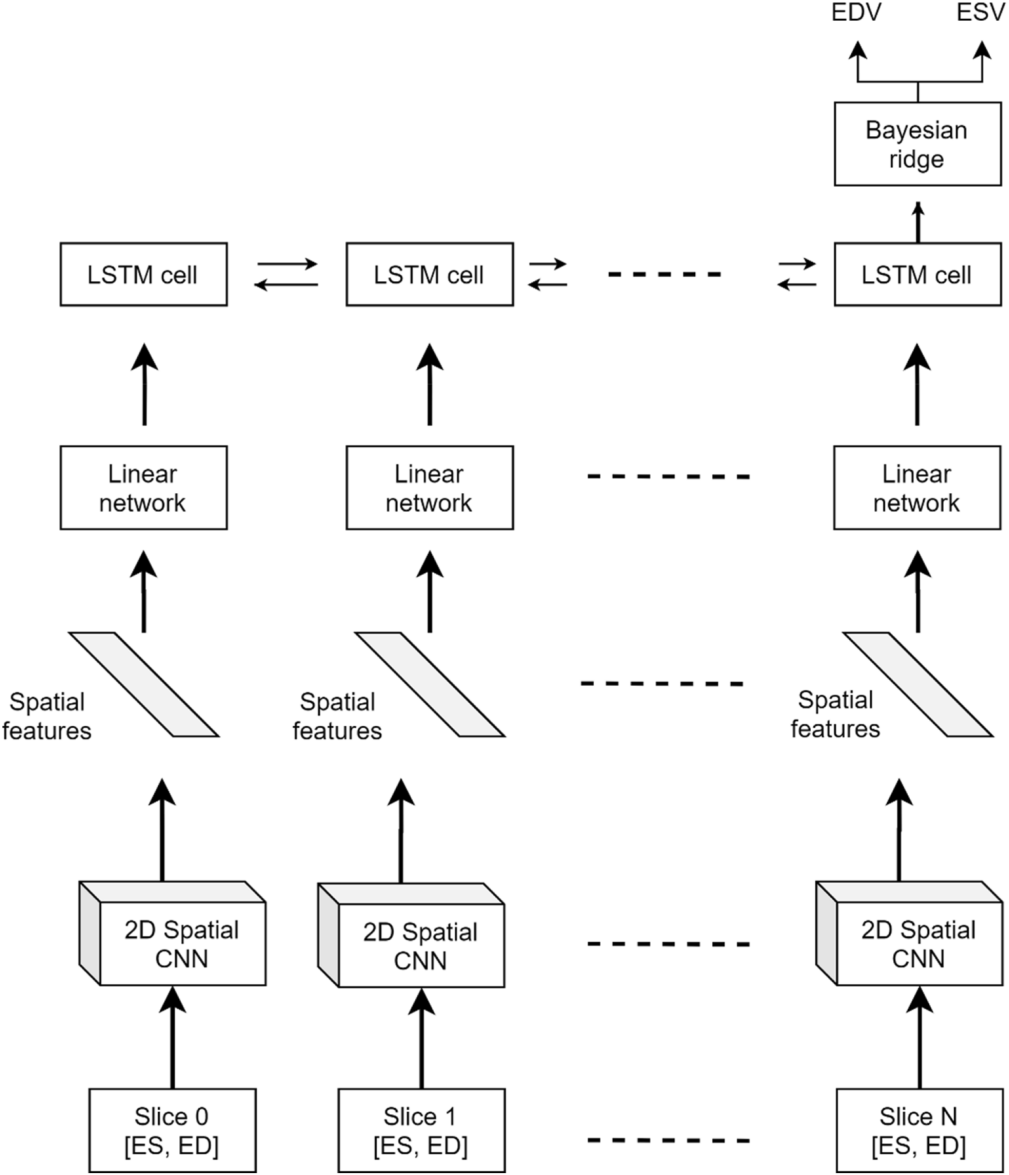
Architecture of the Spatial Feature Encoding Recurrent network for Abstracting high-level patient features (SFERA) model. The model takes as input a SAX stack of variable number of slices which comprise individual ED and ES frames. The network outputs the ESV and ESV, which are subsequently used to compute the ejection fraction.

We designed a custom deep neural network capable of processing a stack of CINE MR slices of variable number of slices and which outputs both EDV and ESV, further used to compute the EF. The architecture of the SFERA network is shown in Fig. 7. The network input is a SAX stack of a varying number of slices, each consisting of one ED and one ES frame concatenated along the channel axis. A 2D residual CNN is employed in the first layers for every (ED, ES) pair. There are five residual blocks building the CNN. Every block consists of multiple 2D convolutional layers, ReLU activation functions, Batch Normalization^28^ and Max Pooling layers. The first convolutional layer outputs 32 channels, and this parameter doubles in value at every convolutional block. Before feeding the resulting features to the LSTM^20^, they are flattened, and a linear network is used to reduce their dimensionality to 128 elements containing spatial information. Then, a bidirectional LSTM^20^ network is applied to correlate the information between these feature vectors, resulting a vector containing both spatial and temporal information. As a final step, a Bayesian ridge regressor is employed to predict the final EDV and ESV volumes. The LSTM^20^ approach enables the proposed model to process a variable length of slices. The training of the SFERA model is performed using the Rectified Adam optimizer and RMSE loss function.

Volume data from the stack of SAX slices is normalized by the distance between slices. We have employed a unity-based normalization to rescale the EDV and ESV values to the range [0, 1]. Only the slices between the basal and the apex planes were retained. After the inference step, the actual ventricular volume (ml) is obtained by scaling the voxel volume estimations output by the network by the original distance between slices.

We used RMSE and Pearson correlation metrics to evaluate the performance of the trained model against the ground truth values for EDV, ESV and EF. The model prediction error was further investigated using Bland–Altman analysis where the confidence interval was defined as mean ± 1.96 SD. Kruskal–Wallis test was used to measure the statistical difference between the RMS errors obtain in the RSO and RSP experiments.

## Supplementary Information

Supplementary Information.
