# Supplementary material for: Improving robustness of automatic cardiac function quantification from cine magnetic resonance imaging using synthetic image data
Source: Sci Rep. 2022 Feb 14;12:2391. doi: 10.1038/s41598-022-06315-3 (PMC8844403; doi:10.1038/s41598-022-06315-3)
Supplement: Supplementary file 1 — Supplementary Information. [file 41598_2022_6315_MOESM1_ESM.docx]

**Supplemental material**

Figure 8 illustrates the learning curves for the three experiments on Dataset 1, all of which were trained for 100 epochs. The learning curves for Dataset 2 were similar. For the last experiment, RSP, where the synthetic model was used for weight initialization, only a couple of epochs were required for local minima convergence, with a relatively small gap between learning curves, indicating a good fit. For the evaluation on the test datasets, the model from the epoch where the best results were obtained on the validation dataset was automatically chosen.

The learning curves for the model trained only on synthetic cases decrease simultaneously to a point of stability, with a relatively small gap between the training and validation curves, indicating a good fit. The loss function of the model trained only on real cases without pretraining, decreases for both the training and the validation data until around epoch 30, and then only the training curve continues to decrease, while the validation curve remains stable. This could point to having insufficient information in the training dataset for learning the predictive function for the validation set. By analyzing the learning curves of the model fine-tuned on real cases, pretrained on synthetic cases, it is noticeable that even though the RMS Error is reduced on the test set compared to the model before finetuning (Table 1), the learning curve on the validation set decreased only marginally. We believe the reason for this to be the validation subset which consisted of real subject datasets with most of the EF values in range 50% – 70%. The original model was already able to predict the EF accurately at the center of the distribution. Based on Table 1, the model trained only on synthetic cases (SSO) nevertheless has a larger RMS Error when tested on real subjects, than the model finetuned on real subjects (RSP). This confirms that the error has significantly reduced as a result of the finetuning step on real patient data.


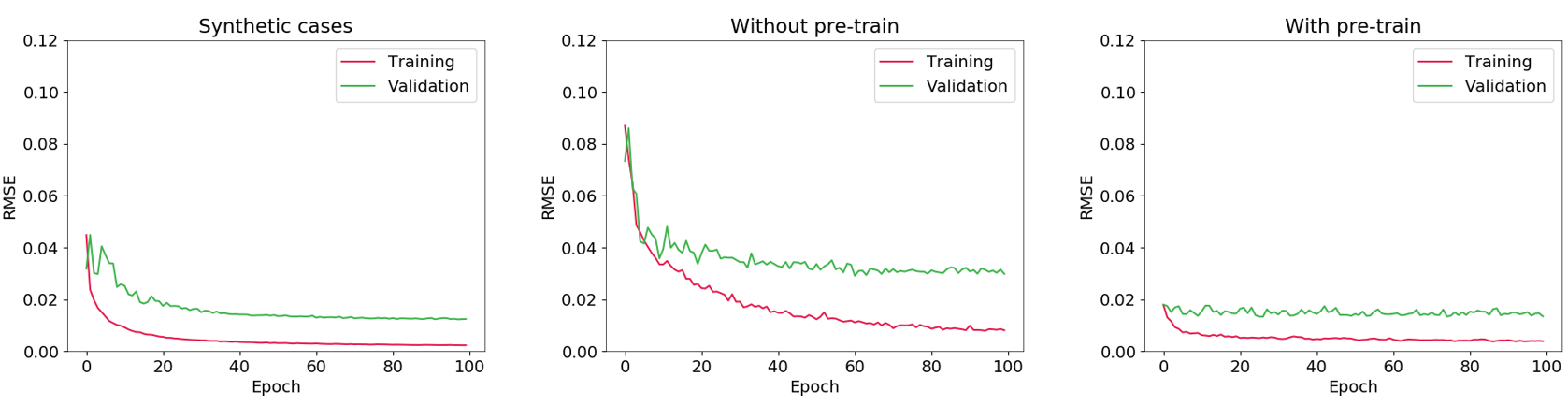
*Figure 8. Learning curves for all three experiments performed on the Dataset 1, from left to right: training on synthetic cases only, on real cases without fine tune, and on real cases with finetune from synthetic model. For the first experiment, the training dataset is not representative enough in relation to the validation dataset, which leads to a large difference between the learning curves and to suboptimal results. For the other two experiments where synthetic data was used, a good fit was obtained.*
